# Fermented Foods: Definitions and Characteristics, Impact on the Gut Microbiota and Effects on Gastrointestinal Health and Disease

**DOI:** 10.3390/nu11081806

**Published:** 2019-08-05

**Authors:** Eirini Dimidi, Selina Rose Cox, Megan Rossi, Kevin Whelan

**Affiliations:** King’s College London, Department of Nutritional Sciences, London SE1 9NH, UK

**Keywords:** kefir, kombucha, sauerkraut, miso, natto, tempeh, soy, kimchi, sourdough, fermented food

## Abstract

Fermented foods are defined as foods or beverages produced through controlled microbial growth, and the conversion of food components through enzymatic action. In recent years, fermented foods have undergone a surge in popularity, mainly due to their proposed health benefits. The aim of this review is to define and characterise common fermented foods (kefir, kombucha, sauerkraut, tempeh, natto, miso, kimchi, sourdough bread), their mechanisms of action (including impact on the microbiota), and the evidence for effects on gastrointestinal health and disease in humans. Putative mechanisms for the impact of fermented foods on health include the potential probiotic effect of their constituent microorganisms, the fermentation-derived production of bioactive peptides, biogenic amines, and conversion of phenolic compounds to biologically active compounds, as well as the reduction of anti-nutrients. Fermented foods that have been tested in at least one randomised controlled trial (RCT) for their gastrointestinal effects were kefir, sauerkraut, natto, and sourdough bread. Despite extensive in vitro studies, there are no RCTs investigating the impact of kombucha, miso, kimchi or tempeh in gastrointestinal health. The most widely investigated fermented food is kefir, with evidence from at least one RCT suggesting beneficial effects in both lactose malabsorption and *Helicobacter pylori* eradication. In summary, there is very limited clinical evidence for the effectiveness of most fermented foods in gastrointestinal health and disease. Given the convincing in vitro findings, clinical high-quality trials investigating the health benefits of fermented foods are warranted.

## 1. Introduction

Fermented foods are defined as “foods or beverages produced through controlled microbial growth, and the conversion of food components through enzymatic action” [1]. Many foods have historically undergone fermentation, including meat and fish, dairy, vegetables, soybeans, other legumes, cereals and fruits. There are several variables in the fermentation process including the microorganisms, the nutritional ingredients and the environmental conditions, giving rise to thousands of different variations of fermented foods. Historically, food fermentation was performed as a method of preservation, as the generation of antimicrobial metabolites (e.g., organic acids, ethanol and bacteriocins) reduces the risk of contamination with pathogenic microorganisms. Fermentation is also used to enhance the organoleptic properties (e.g., taste and texture), with some foods, such as olives, being inedible without fermentation that removes bitter phenolic compounds.

There are two main methods through which foods are fermented. Firstly, foods can be fermented naturally, often referred to as “wild ferments” or “spontaneous ferments”, whereby the microorganisms are present naturally in the raw food or processing environment, for example sauerkraut, kimchi, and certain fermented soy products. Secondly, foods can be fermented via the addition of starter cultures, known as “culture-dependent ferments”, for example kefir, kombucha and natto [2]. One method of performing a culture-dependent ferment is “backslopping”, in which a small amount of a previously fermented batch is added to the raw food, for example sourdough bread [1]. Starters used to initiate fermentation can be either natural (e.g., backslopping), or selected commercial starters to standardize the organoleptic characteristics of the final product [3].

Fermented foods hold a firm place in cuisine from almost every culture in the world. In the West, there has been a surge in popularity of fermented foods in recent years, major reasons including the proposed health benefits of fermented foods and surging interest in gastrointestinal health. There are several mechanisms through which fermented foods may exert beneficial effects in health and disease.

Firstly, they contain potentially probiotic microorganisms, such as lactic acid bacteria [1]. In general, most fermented products have been found to contain at least 10^6^ microbial cells per gram, with concentrations varying depending on several variables such as the product’s region, age and time at which the products are analysed/consumed [2]. The surrounding food matrix appears to play an important role in the survival of probiotic strains via its buffering and protective effect against gut conditions (e.g., low pH, bile acids) [4]. Indeed, a number of studies have shown that microorganisms from fermented foods can reach the gastrointestinal tract, this is likely to differ across products, and their presence in the gut appears to be transient [5]. Nonetheless, these microorganisms may still have the potential to exert a physiological benefit in the gut, through competition with pathogenic bacteria and the production of immune-regulatory and neurogenic fermentation by-products [6]. Secondly, fermentation-derived metabolites may exert health benefits. For example, lactic acid bacteria (relevant to both dairy and non-dairy fermented foods) generate bioactive peptides and polyamines with potential effects on cardiovascular, immune and metabolic health [7]. Thirdly, fermentation may convert certain compounds to biologically active metabolites. For example, lactic acid bacteria can convert phenolic compounds (such as flavonoids) to biologically active metabolites [8]. Fourthly, food components found in fermented foods, such as prebiotics and vitamins, may also exert health benefits [1,9]. Lastly, fermentation can reduce toxins and anti-nutrients, for example, fermentation of soybeans may reduce phytic acid concentrations [10], and sourdough fermentation can reduce the content of fermentable carbohydrates (e.g., fermentable oligosaccharides, disaccharides, monosaccharides and polyols, FODMAPs), which may increase the tolerance of these products in patients with functional bowel disorders such as irritable bowel syndrome [11].

This review aims to define and characterise common fermented foods, their mechanisms of action (including impact on the microbiota), and the evidence for effects on gastrointestinal health and disease in humans. The evidence for the effects of yoghurt and cheese on human health has been extensively reviewed elsewhere [12,13] and, therefore, this review will focus specifically on kefir and the major non-dairy fermented foods: kombucha; sauerkraut; tempeh; natto; miso; kimchi; and sourdough bread (Table 1).

## 2. Kefir

Traditional kefir, which originates from the Caucasus Mountains, is a fermented milk drink with a creamy texture, sour taste and subtle effervescence. It is produced by adding a starter culture termed “kefir grains” to milk. Kefir grains consist of symbiotic lactose-fermenting yeasts (e.g., *Kluyveromyces marxianus*) and non-lactose fermenting yeasts (e.g., *Saccharomyces cerevisiae*, *Saccharomyces unisporus*), as well as lactic and acetic acid producing bacteria, housed within a polysaccharide and protein matrix called kefiran [34]. Lactic acids, flavour-generating components (e.g., acetaldehyde), ethanol and carbon dioxide are all by-products of fermentation and contribute to the organoleptic properties of kefir [35]. A dairy-free version of kefir also exists, called water kefir, which is a fermented beverage made of water, sugar and water kefir grains, which contains bacteria and yeasts, albeit different to the traditional kefir starter cultures. There is very limited evidence on water kefir and, therefore, this section focuses on traditional kefir only.

A wide range of microbial species have been identified in kefir grains, commonly including *Lactobacillus brevis*, *L. paracasei*, *L. helveticus*, *L. kefiranofaciens*, *L. plantarum*, *L. kefiri*, *Lactococcus lactis*, *Streprotcoccus thermophiles*, *Acetobacter lovaniensis*, *Acetobacter orientalis*, *Saccharomyces cerevisiae*, *S. unisporus*, *Candida Kefyr*, *Kluyveromyces marxianus* and *Leuconostoc mesenteroides* [14,36,37]. Following fermentation, the microbial composition of kefir may change. For example, although not a predominant Lactobacillus species in the kefir grain starter culture, *L. kefiri* can represent 80% of all *Lactobacillus* species in the final fermented beverage [14,36]. The Food and Agriculture Organisation (FAO) and the World Health Organisation (WHO) suggest that kefir grains should contain a minimum 10^7^ colony forming units (CFU)/g micoorganisms and the final product should contain at least 10^4^ CFU/g of yeast [38].

Several in vitro studies have investigated kefir’s antimicrobial activity, which is attributed to competition with pathogens for available nutrients, as well as the production of organic acids, bacteriocins, carbon dioxide, hydrogen peroxide, ethanol and diacetyl [39]. In vitro studies have shown that kefir exhibits antimicrobial activity against *Candida albicans*, *Salmonella typhi*, *Salmonella enterica*, *Shigella sonnei*, *Escherichia coli*, *Bacillus subtilis*, *Enterococcus faecalis* and *Staphylococcus aureus* [40,41].

Fermentation-derived bioactive peptides produced from casein have been shown to stimulate the immune system in animal models [42], while kefiran reduced ovalbumin-induced cytokine production in a murine asthma model [43]. In vitro and animal studies have also suggested potential anti-oxidative, anti-hypertensive, anti-carcinogenic and cholesterol-and glucose-lowering effects of kefir [44,45,46,47,48,49].

The impact of kefir and its constituent microorganisms on the gut microbiota has been investigated in several in vitro, animal and human studies. Although not yet confirmed in vivo, several strains isolated from kefir have been shown to adhere to human enterocyte-like Caco-2 cells, indicating a potential ability to colonise the human gut [50]. Kefir, or its constituent strains, have also been shown to have a considerable impact on the gut microbiota population with increases in *Lactobacillus*, *Lactococcus* and *Bifidobacterium* concentrations, and reductions in *Proteobacteria* and *Enterobacteriaceae* concentrations, being demonstrated in numerous animal studies [51,52,53]. Furthermore, one study found higher concentrations of *Firmicutes*, *Bacteroidetes* and *Prevotella*, as well as a higher stool weight and stool water, in mice administered *Lactobacillus kefiranofaciens* (but not kefir *per se*), a common strain found in kefir grains, compared to control [52]. In addition, a study showed that kefiran increased stool weight and moisture in rats, in a dose-responsive manner, compared to control, suggesting a potential beneficial effect in constipation [54]. The abundance of yeasts in the gastrointestinal tract is also altered following kefir consumption; a study in a high-fat diet-induced obese mouse model showed that those who ingested 0.2 mL kefir had significantly greater number of stool total yeasts and *Candida kefyr* compared to control mice [55]. In humans, a study in 45 people with inflammatory bowel disease showed that a kefir-specific strain, *Lactobacillus kefiri*, was identified in most participants’ faeces 4 weeks following 800 mL/day kefir consumption, and a significant increase in total stool *Lactobacillus* abundance was found compared to control (no kefir) in patients with Crohn’s disease [56].

### Kefir in Gastrointestinal Health and Disease

Several studies have been carried out in humans investigating the effect of kefir consumption on gastrointestinal function and dysfunction. Kefir has been suggested to be well tolerated by people with lactose malabsorption since it contains β-galactosidase expressing bacteria (e.g., *Kluyveromyces marxianus*), which hydrolyses lactose, thus reducing lactose concentrations in the drink. Kefir contains 60% more β-galactosidase than plain yoghurt, while a 30% reduction in lactose content has been shown in kefir compared with unfermented milk [57]. Despite reportedly greater β-galactosidase concentrations in kefir than yoghurt, a small cross-over randomised controlled trial (RCT) in 15 people with lactose malabsorption showed that although kefir produced a significantly lower breath hydrogen concentration compared to milk, it was similar following kefir and plain yoghurt, suggesting that kefir and plain yoghurt improved lactose digestion to a similar degree [57]. Kefir also led to a significantly lower flatulence severity compared to milk, but no differences were seen for flatulence frequency, abdominal pain and diarrhoea [57]. Overall, this study suggests kefir results in lower flatulence severity than milk, and is as well tolerated as yoghurt, in people with lactose malabsorption.

Several non-randomised studies have also explored the impact of kefir in constipation [58,59,60] (Table 2). A non-randomised cross-over study in 42 hospitalised patients with constipation and mental and physical disabilities showed that 6 g of lyophilized kefir had no impact on laxative use, stool consistency and stool volume compared to control powdered milk, however the number of patients not requiring any laxatives was higher 12 weeks following the kefir intervention compared to baseline (Table 2) [59]. Another small non-randomised, uncontrolled trial in 20 people with functional constipation showed that 500 mLs of kefir for 4 weeks significantly increased stool frequency, improved bowel satisfaction score and reduced gut transit time compared to baseline [60]. Considering the small sample and limitations in study design (difference in kefir form, no randomisation, uncontrolled, limited use of validated procedures), further high-quality trials are required to establish the impact of kefir on constipation.

In a RCT, kefir led to a significantly greater increase in stool *Lactobacillus* concentration and in blood haemoglobin concentration in patients with Crohn’s disease (*n* = 10) compared to control (*n* = 20) (Table 2); no change was shown however in ulcerative colitis (*n* = 15) [56].

Another double-blind RCT investigated the impact of 500 mL/day kefir, compared to 250 mL/day milk, on *Helicobacter pylori* eradication rates in patients with dyspepsia and diagnosed *H. pylori* infection who were taking a triple antibiotic therapy for 2 weeks [61]. The study found that the rate of *H. pylori* eradication was significantly higher in the kefir group (78%) compared to the control group (50%; *p* = 0.026) [61]. The occurrence of diarrhoea, abdominal pain and nausea were also significantly lower in the kefir group compared to control, suggesting kefir may be beneficial adjunct therapy during treatment for *H. pylori* infections (Table 2).

Another double-blind RCT in 125 children prescribed antibiotics for upper respiratory infections examined the effect of 150 mL/day kefir for 14 days, compared to 150 mL/day heat-treated kefir, in preventing antibiotic-associated diarrhoea [62]. This study showed that kefir with live microorganisms did not improved the rates of antibiotic-associated diarrhoea compared to control (relative risk 0.82, 95% confidence interval (CI) 0.54–1.43), and no differences were found for any of the symptoms assessed, including abdominal pain, loose stools, constipation and vomiting (Table 2) [62].

There are currently no RCTs investigating the effects of kefir in functional bowel disorders.

To conclude, there is evidence from RCTs demonstrating kefir may be beneficial for lactose malabsorption, and *H. pylori* eradication. However, an important limitation of kefir studies is that each batch may consist of different microorganisms. This may explain some of the heterogeneous findings. Further high-quality RCTs are needed to establish the impact of kefir on the gut microbiota and its impact on other gastrointestinal disorders, such as constipation.

## 3. Kombucha

Kombucha is a fermented tea beverage reported to have originated in Northeast China in around 220 BC and consumed extensively during the Qin Dynasty. Similar fermented tea beverages subsequently became popular in Russia and Eastern Europe [63]. In modern societies, a range of kombucha beverages are available commercially, although the microbial and metabolite composition of these products along with methods of production are rarely reported [15].

Traditional kombucha is produced through aerobic fermentation of black tea (green tea may also be used) and white sugar by a combination of bacteria and yeast, known as the symbiotic culture of bacteria and yeast (SCOBY). The yeast convert sucrose to ethanol (in addition to organic acids and carbon dioxide) which acetic acid bacteria convert to acetaldehyde and acetic acid [64]. The microbial and metabolite composition of kombucha varies according to the exact composition of the SCOBY, the type and concentration of tea and sugar [65,66], oxygen concentrations, fermentation time [67,68], temperature [67,69] and storage duration [65]. The low pH of kombucha, owing mainly to the production of high concentration of acetic acid, has been shown to prevent the growth of pathogenic bacteria such as *Helicobacter pylori*, *Escherichia coli*, *Salmonella typhimurium* and *Campylobacter jejuni* [70]. Even at neutral pH and after thermal denaturation, kombucha was able to inhibit the growth of pathogens in vitro, suggesting that compounds other than acetic acid exert antimicrobial effects [70].

The bacterial and fungal species constituting the SCOBY typically include acetic acid bacteria (*Acetobacter*, *Gluconobacter*), lactic acid bacteria (*Lactobacillus*, *Lactococcus*) and yeasts (*Saccharomyces, Zygosaccharomyces*) [15,63,71]. Studies utilising high-throughput sequencing analysis have demonstrated that following fermentation, *Candida* and *Zygosaccharomyces* genera are the predominant yeasts in kombucha [15,16], while *Komagataeibacter*, *Lyngbya*, *Gluconobacter*, *Lactobacilli* and *Bifidobacteria* are the most abundant bacterial genera.

Kombucha has been shown to exert effects in animal studies on blood glycaemia [72], oxidative stress [73], diabetes-induced weight loss [74], chemically-induced nephrotoxicity [75], hypercholesterolaemia [72,76] and indomethacin-induced gastric ulceration [77]. Compounds hypothesised to play a role in these beneficial effects include d-saccharic acid-1,4-lactone (DSL). This is produced by *Gluconobacter* during fermentation [78,79], and in rats, inhibits oxidative stress and diabetes-induced renal damage [80] and acetaminophen-induced hepatic injury [81]. Nonetheless, there is no human data on DSL to confirm this proposed mechanism of action. Polyphenol and flavonoid content of tea increases with fermentation [15]. Furthermore, in vitro superoxide radical scavenging ability, reducing ability and total phenolic compound concentration increases during kombucha fermentation [82].

Although kombucha is a rich source of acetic acid and lactic acid bacteria and yeasts [15], there are no published studies exploring the effect of kombucha consumption on the gastrointestinal microbiota composition or function in either animals or humans. Interestingly, kombucha has been shown to have antimicrobial effects in vitro [83,84]. It is currently unknown whether the proposed physiological effects of kombucha are mediated by the gastrointestinal microbiota or other direct immunological pathways.

Despite evidence of physiological effects of kombucha consumption in animals, the effects in humans remain largely unknown. A recent systematic review [85] did not identify any RCTs of kombucha on gastrointestinal disorders, including any of the functional bowel disorders.

To conclude, there are no studies of the effects of kombucha on gastrointestinal health and microbiota in humans.

## 4. Sauerkraut

Sauerkraut is one of the most common forms of preserved cabbage originating in the 4th century BC. Sauerkraut is eaten frequently in Germany, but also in other European and Asian countries and the United States [86]. Sauerkraut is produced from a combination of shredded cabbage and 2.3%−3.0% salt, which is left to undergo spontaneous fermentation, generally involving *Leuconostoc* spp., *Lactobacillus* spp., and *Pediococcus* spp. The low pH of the final product results in a preserved cabbage [87].

Sauerkraut (homemade and shop-bought) has been shown, through culture-dependent techniques, to contain *Bifidobacterium dentium*, *Enterococcus faecalis*, *Lactobacillus casei*, *Lactobacillus delbrueckii*, *Staphylococcus epidermidis*, *Lactobacillus sakei*, *Lactobacillus curvatus*, *Lactobacillus plantarum*, *Lactobacillus brevis*, *Weissella confusa*, *Lactococcus lactis* and *Enterobacteriaceae* [17,18,88]. Adding a starter culture of *Lactobacillus casei* 11MZ-5-1 produced a sauerkraut containing predominantly *Lactobacillus* and *Lactococcus*, compared to spontaneous sauerkraut which, along with Lactococcus and Lactobacillus, also contained significant *Enterobacter* and *Pseudomonas* and was more variable in microbial composition [89]. Sauerkraut has also been shown to predominantly contain *Leuconostoc* and *Lactobacillus* spp. [18,19,90,91]. Certain Lactobacillus species isolated from sauerkraut demonstrate probiotic potential, with tolerance to low pH, adherence to Caco-2 cells and antimicrobial activity against pathogens in vitro [92]. *Lactobacillus paracasei* HD1.7, commonly found in sauerkraut, has been shown to produce a broad-spectrum bacteriocin that may play a role in sauerkraut preservation [93].

Oral administration of sauerkraut juices in Wistar rats led to increased activity of glutathione S-transferase (GST) and NAD(P)H:quinone oxidoreductase 1 (NQO1), key liver and kidney detoxifying enzymes [94]. Certain lactic acid bacteria contained in sauerkraut generate conjugated linoleic acid [95], for which there is evidence of anti-carcinogenic and anti-atherosclerotic activity in animals [96,97]. Furthermore, *Lactobacillus plantarum* P2 isolated from sauerkraut significantly induced TNF-α and IL-12 expression and prevented adhesion and invasion of Caco-2 cells by *Salmonella* enteritidis [98]. Sauerkraut contains glucosinolate breakdown products including kaempferol, (a flavonoid) isothiocyanates, indole-3-carbinol, goitrin, allyl cyanide and nitriles [99]. The relevance of such phytochemicals to human health is unclear, however kaempferol has been shown to have radical scavenging activity, to protect from oxidative damage and to attenuate cytokine-induced reactive oxygen species in vitro [100]. Isothiocyanates have been shown to have antimicrobial properties, preventing the growth of a range of species, including *E. coli*, *C. difficile*, *C. jejuni* and *C. perfringens* [101].

Sauerkraut is one of the few fermented foods for which there is a clinical trial in functional bowel disorders. A randomised double-blind trial compared the effects of sauerkraut containing viable lactic acid bacteria (LAB) on gastrointestinal symptoms and microbiota in 58 patients with irritable bowel syndrome (IBS) of any subtype diagnosed using Rome III criteria [18]. Patients were randomised to consume 75 g/day pasteurised (control) or unpasteurised (intervention) sauerkraut containing LAB for 6 weeks. There was a significant reduction in IBS Severity Scoring System (IBS-SSS) score between baseline and end of trial in both study groups, however there was no difference in symptoms between the diet groups; 16S rRNA sequencing revealed no difference in microbiota composition between study groups or between baseline and end of trial in either group (Table 3). This may suggest that the perceived health benefit of sauerkraut is independent of the live microbes. A limitation of this study is the per protocol analysis in that only patients who completed the study (*n* = 34) were included in the analysis of the primary outcome. Furthermore, because there was no raw cabbage arm, it is not possible to determine whether improvement in gastrointestinal symptoms was related to the fermentation-derived products or the cabbage itself.

Another study in Chinese participants suggested larger amount of sauerkraut may in fact be associated with poor health outcomes in gastrointestinal cancers. This case-control study found that the highest compared to the lowest quintile of sauerkraut intake was associated with a greater risk of laryngeal cancer (odds ratio (OR) 7.27) [102]. One possible mechanism may relate to the high salt content of sauerkraut, although another case-control study of dietary risk factors for laryngeal cancer in China showed no associations with salt-preserved vegetables [103]. Similarly, the high potassium content of sauerkraut is thought to counter the hypertensive effects of added salt.

Taking the limited evidence for sauerkraut into account, one trial indicates that both pasteurised and unpasteurised sauerkraut reduced IBS severity, this effect does not appear to be mediated by gastrointestinal microbiota. Further studies are required to elucidate the mechanisms of this effect on gastrointestinal symptoms. There is little evidence for effects of sauerkraut on other health conditions. 

## 5. Fermented Soy Products (Tempeh, Natto, Miso)

The first known fermented soy products originated in China and Japan, including fermented black soybean and red fermented tofu [107]. There are many fermented soybean products from different parts of Asia, including tempeh, natto, miso, sufu, douche, soy sauce and doenjang. This review will focus on tempeh, natto and miso.

### 5.1. Tempeh

Tempeh is a traditional Indonesian food produced by fermenting boiled and dehulled soybeans with a starter culture of *Rhizopus oligoporus* fungal species at room temperature for 35–37 hours [107,108]. This produces a soft white cake with a chewy texture and mushroom-like flavour. The microbial composition of tempeh varies according to variations in production [109]. Tempeh contains lactic acid bacteria [2,110], *Enterococcus faecium* [110], and Rhizopus filamentous fungi. Fermentation of soybeans has been shown to reduce concentrations of protease inhibitors, phytic acid and phenols [10], antinutritional factors that are high in raw soybeans, which may relate to phytases expressed by Rhizopus species in tempeh [111].

In Sprague–Dawley rats, stool Bacteroidetes, Firmicutes, *Clostridium leptum* and *Bacteroides fragilis* abundance increased following tempeh supplementation compared to rats fed non-fermented soybeans [112]. Application of soybean and bean tempeh to human microbiota in an in vitro gut simulator model increases *Bifidobacterium*, *Lactobacillus*, *Escherichia coli* and *Enterococcus* abundance [113]. In an open-label uncontrolled study of 10 healthy human volunteers, tempeh consumption led to greater stool *Akkermansia muciniphila* abundance and immunoglobulin A concentrations, suggesting that tempeh may influence gut microbiota in humans [114]. However, a larger, controlled study is needed to establish the effects of tempeh on gastrointestinal microbiota composition.

Fermented soy products have been proposed to have beneficial effects on health, including purported “anti-carcinogenic”, “anti-diabetic”, “antioxidant”, “anti-inflammatory” and “anti-hyperlipidaemic” effects, although much of the existing evidence is limited to in vitro and animal studies [107]. Tempeh has been associated in vitro with greater free-radical and superoxide scavenging ability than unfermented soybeans [115], which may relate to changes in polyphenol content and digestibility in soybeans following fermentation [116,117].

To date, there are no RCTs of the effects of tempeh consumption in humans. Proposed health effects listed above require investigation in human trials.

### 5.2. Natto

Natto is a traditional Japanese fermented soybean, of which Itohiki-Natto is the most commonly consumed [108]. Natto is produced through fermentation of cooked yellow soybeans with *Bacillus subtilis* var. natto. This produces a viscous food with a distinct flavour and strong odour [118]. Natto characteristics vary according to soybean steaming time, relative humidity, fermentation time and temperature [108]. The fermentation of Natto produces a number of bioactive factors, including nattokinase, bacillopeptidase F, vitamin K_2_ and dipicolinic acid [108]. Furthermore, the quantity of the isoflavone genistein, with purported associations with metabolic and inflammatory disorders and carcinogenesis [119], is greater in Natto compared to unfermented soy products [120]. A peptide with antibacterial activities against *Streptococcus pneumoniae* and *Bacillus subtilis* has been isolated from Natto [121], with potential clinical importance in treating *S. pneumoniae* infections, although this has not been investigated in humans.

Nattokinase is an enzyme of the subtilisin family produced by *Bacillus* subtilis var. natto [122], and can be isolated from Natto [123]. Nattokinase has direct in vitro [123] and in vivo [124] fibrinolytic activity, in addition to increasing tissue plasminogen activator [125] and reducing platelet aggregation [126]. Anti-thrombotic and anti-hypertensive activities of nattokinase have been demonstrated in small RCTs in humans [127,128].

There is limited evidence on the effect of Natto on the human GI microbiota. Consumption of Natto-containing miso soup for two weeks led to increased stool *Bacilli* and *Bifidobacteria* and decreased Clostridia and Enterobacteriaceae in 8 healthy volunteers [104]. Furthermore, stool short-chain fatty acids increased, and ammonia and sulphide declined. However, it is impossible to separate the effect of the miso soup (also a fermented soy product) from possible effects of Natto. In individuals with 3–5 bowel movements per week, consumption of 50 g/day *Bacillus subtilis* K-2 containing Natto for two weeks resulted in greater stool frequency and proportion of stool *Bifidobacteria* compared to consumption of 50 g/day of boiled beans [106], although only the abstract is available and no sample size is provided.

To date, there is limited evidence from RCTs suggesting Natto might positively influence stool frequency in patients with infrequent bowel motions and influences gastrointestinal microbiota. However, these require confirmation in high quality trials.

### 5.3. Miso

Miso is a traditional Japanese paste of fermented soybean used to make miso soup. Miso is produced by fermenting soybeans with ‘Koji’, produced from a mould *Aspergillus oryzae*, although *Saccharomyces cerevisiae* and lactic acid bacteria may additionally be used. As with other fermented soy foods, miso production varies greatly in terms of ingredients, temperature and fermentation time, salt concentration and the strain of *A. oryzae* used.

A microbial analysis of miso at different time points following the start of fermentation revealed *Bacillus subtilis*, *Bacillus amyloliquefaciens*, *Staphylococcus gallinarum* and *Staphylococcus kloosii* to be present during fermentation, with only the *Bacillus* species remaining in the final product [129]. A range of miso samples have also been shown to contain *Lactococcus* sp. GM005, which produces a bacteriocin with strong antibacterial activity that inhibits the growth of a range of bacteria, including *Bacillus subtilis*, *Pediococcus acidilactici* and *Lactobacillus plantarum* [130,131].

There is little evidence on the effect of miso intake on gastrointestinal disorders. One cross-sectional study reported an inverse relationship between miso soup intake and subjective gastro-oesophageal reflux disease, functional dyspepsia and reflux scores when adjusted for other dietary factors [132]. This association was hypothesised to relate to histidine, glutamate and aspartate found in miso soup, although no animal or human studies have investigated this mechanism to date.

The high soy intakes in China and Japan have historically been hypothesised to contribute to the relatively low rates colon and prostate cancers in these countries [133]. One proposed mechanism to support this hypothesis is the high concentrations of isoflavones genistein and daidzein found in soybeans [134]. Genistein is structurally similar to oestrogen and may influence breast cancer risk through oestrogen receptor binding, which has been demonstrated in vitro [135,136]. Further in vitro studies have demonstrated that genistein may exert effects on cancer risk through promoting cell-cycle arrest [137] inducing apoptosis [138] and reducing cancer cell migration [139]. Genistein and daidzein may be higher in fermented soybean products (miso and natto) compared to unfermented products such as soy milk and tofu [120,140,141], as previously discussed. Numerous Japanese cohort studies have investigated associations between miso intake and cancer risk. These studies are limited in the assessment of dietary intake (food frequency questionnaires, often with limited intake responses) and the presence of a multitude of possible confounding factors. With these methodological limitations in mind, cohort studies have observed inverse associations between frequent miso soup intake and stomach cancer risk in Japanese men [142]. In contrast, cohort and case-control studies have shown positive correlations between frequent miso soup intake and single and multiple stomach cancers in Japanese adults [143]. Furthermore, some cohort studies have shown no association between miso soup intake and risk of various types of cancer [144,145].

There are currently no RCTs investigating the effects of miso in functional bowel disorders. There is therefore limited evidence for the effects of miso on gastrointestinal conditions and microbiota. There are several observational studies demonstrating associations between miso intake and the risk of stomach cancer, however the strength and direction of these associations remains unclear.

## 6. Kimchi

Kimchi, which originates from Korea, is a term used for a group of salted and fermented vegetables. It consists of Chinese cabbage and/or radishes, and various flavouring ingredients (e.g., chili, pepper, garlic, onion, ginger), seasonings (e.g., salt, soybean sauce, sesame seed), and other additional foods (e.g., carrot, apple, pear, shrimps) [20]. To produce kimchi, the cabbage is brined and drained, then the rest of the seasonings, spices and food products are added and mixed with the cabbage, and finally, fermentation takes place (134). The fermentation occurs spontaneously by the microorganisms naturally found on the cabbage and foods included in the mixture, although starter cultures may be used for commercial production of kimchi [20].

Prior to fermentation, the kimchi mix contains a variety of different bacterial species within the Leuconostoc, Lactobacillus, Pseudomonas, Pantoea and Weissella genera [21] (Table 1). However, once fermentation has started, the bacterial diversity decreases and the bacterial community is rapidly dominated by the genus Leuconostoc within only three days of fermentation [21]. Within this genus, Leuconostoc citreum is the most abundant species prior to fermentation, but it is present in only a minor proportion after three days of fermentation, at which time point Leuconostoc gasicomitatum and Leuconostoc gelidum become dominant [21]. As kimchi can comprise a variety of ingredients, the microbial composition varies depending on the type and amount of the foods included. For example, a higher Lactobacillus concertation has been found when kimchi contains a higher garlic quantity [146], while the addition of red pepper powder leads to higher Weissella and lower Leuconostoc and Lactobacillus proportions [147]. Several archaea (e.g., Halococcus, Natronococcus) and yeast (e.g., Saccharomyces, Candida, Trichosporon) genera have also been identified in commercially available kimchi [22] (Table 3). An animal study showed that kimchi consumption, which contained *Leuconostoc mesenteroides* DRC 0211, may exhibit potential weight control properties in mice via reducing hepatic mRNA expression of adipogenesis-related genes and inflammation-related monocyte chemotactic protein-1 and interleukin-6 in epididymal fat tissue [148]. Reductions in serum total cholesterol, triglycerides, low-density lipoprotein cholesterol levels and atherogenic index have also being demonstrated in rats following consumption of kimchi fermented by *Leuconostoc kimchi* GJ2 [149]. A human study demonstrated that consumption of kimchi fermented for 8 weeks led to changes in expression of genes related to metabolic pathways and immunity [150]. In a mouse colitis model, *Lactobacillus paracasei* LS2, a strain isolated from kimchi, decreased cytokine production, myeloperoxidase activity, and the number of macrophages and neutrophils in the lamina propria lymphocytes, suggesting a potential anti-inflammatory effect [151]. Anti-carcinogenic properties have also been attributed to kimchi with an in vitro study demonstrating inhibition of gastric cancer cell growth [152]. Notably, as kimchi comprises a variety of ingredients, its impact on the gut microbiota and health is thought to result from a synergic effect of the microorganisms it contains, as well as the nutrient content (e.g., phytochemicals, fibre, vitamins) of the foods used in the preparation. For example, antimicrobial and antioxidant effects have also been attributed to food constituent of kimchi, such as red pepper seeds and garlic [153,154].

Several studies have investigated the impact of kimchi on the gut microbiota. A study on diet-induced obesity murine models showed that *Lactobacillus plantarum* HAC01, isolated from kimchi, resulted in a higher *Adlercreutzia* and lower *Bacteroides*, *Mucispirillum* and *Ruminococcus* proportions compared to control mice [155]. In humans, a non-randomised study of 6 people in South Korea showed that consumption of 300 g/day kimchi for 4 weeks increased stool concentrations of *Lactobacillus* and *Leuconostoc*, and decreased stool pH, compared to 60 g/day of kimchi [105]. Similar findings have also been identified in several other non-randomised human studies [156,157]. In a RCT of fermented compared to fresh (unfermented) kimchi in 24 women with obesity, women who were randomised to receive 180 g/day fermented kimchi for 8 weeks showed a decrease in *Blautia* abundance and increases in *Prevotella* and *Bacteroides* abundance compared to baseline, but both groups (fermented and unfermented kimchi) experienced increases in *Proteobacteria* and *Actinobacteria* abundance [150]. Another RCT compared two different kimchi preparations made of different ingredients and quantities; kimchi I contained baechu cabbage, radish, red pepper powder, green onion, garlic, ginger, anchovy juice, and sugar, while kimchi II contained organic baechu cabbage, radish, red pepper powder, green onion, garlic, ginger, sugar, mustard leaf, Chinese pepper, pear, *Lentinus edodes* juice and sea tangle juice, mistletoe extract powder, and 10^6^ CFU/g of *Lactobacillus plantarum* PNU [158]. Both kimchi groups resulted in an increased abundance of short-chain fatty acid producing *Faecalibacterium*, *Roseburia*, and *Phascolactobacterium*, and reduced *Clostridium* and *Escherichia coli* compared to baseline. Although no direct comparisons between the two kimchi groups were made, kimchi I was shown to increase the relative abundance of *Actinobacteria* and decrease that of *Proteobacteria* compared to baseline, whereas kimchi II had the opposite effect suggesting that different types and quantities of ingredients in kimchi may impact the microbiota differently [158].

With regards to the effects of kimchi on gastrointestinal health and disease, a very small study attempted to investigate its impact on *H. pylori* eradication. Six people with *H. pylori* infection consumed 300 g (high dose) or 60 g (low dose) of kimchi for 4 weeks in a non-randomised trial [105]. *H. pylori* infection, assessed via ^13^C urea breath test, was not eradicated in any of the six participants at the end of the intervention [105] (Table 2). Kimchi has also been investigated with regards to its association with gastric cancer in epidemiological studies. A number of epidemiological studies have shown an increased risk of gastric cancer in the Korean population with higher kimchi intake (OR 2.2, 95% CI 1.3–3.8), which has been suggested to be due to its nitrite, nitrate and salt content [159,160]. However, a case control study of 136 patients diagnosed with gastric cancer and 136 healthy controls showed that different types and preparations of kimchi were associated with different levels of gastric cancer risk; for example, moderate baiechu kimchi (prepared with salted Chinese cabbage) intakes were associated with a lower gastric cancer risk (OR 0.5, 95% CI 0.3–0.9), while moderate intakes of kkakduki (prepared with salted radish) were associated with a higher gastric cancer risk (OR 2.0, 95% CI 1.03–3.8) [161]. These differences could potentially be attributed to the different food and nutrient composition and preparation methods of the different types of kimchi.

There are currently no RCTs investigating the effects of kimchi in functional bowel disorders.

To conclude, there is preliminary evidence that kimchi may have an impact on the gut microbiota composition. No evidence exists to date on the impact of kimchi on gastrointestinal health and disease, while the association between kimchi consumption and gastric cancer risk warrants further research.

## 7. Sourdough Bread

The sourdough starter culture is produced through the fermentation of flour by lactic acid bacteria and yeasts, that originate from the flour and surrounding environment. Making the sourdough starter takes on average seven days and involves replenishing the microbes with fresh flour and water daily. Once the starter is ready, a small portion is added to the sourdough base ingredients to initiate the sourdough fermentation process—this method is commonly referred to as “backslopping” [24,160]. Unlike standard bread, which is produced through a rapid yeast-only fermentation process, the symbiotic sourdough fermentation of both bacteria and yeast is thought to improve bread quality, including texture, flavour, nutritional content and shelf-life, and replace additives [24]. During fermentation, microbial and enzymatic-led conversions of cereal carbohydrates, proteins, lipids and phenolic compounds occur [24,162]. The microorganisms’ and enzymes’ activity are interlinked; for example, lactic acid bacteria result in a pH reduction, modulating the activity of cereal enzymes and solubility of substrates (e.g., gluten), and in turn the enzymes can provide substrates to allow the growth of microorganisms [24].

The microbial content of the sourdough starter depends on the traditional practices used and, therefore, not only the taste and texture, but the nutritional profile of the final product can vary considerably [25]. In general, several species within the *Lactobacillus*, *Leuconostoc*, *Weissella*, *Pediococcus* and *Streptococcus* genera have been identified in sourdough starters [162]. *Lactobacillus* species are the most prevalent, and *Lactobacillus sanfransiscensis* is a key bacterium isolated from most starters [25]. *Saccharomyces cerevisiae* is the most abundant yeast species, followed by *Candida milleri*, *C. humilis*, *Saccharomyces exiguous* and *Issatchenkia orientalis*. Only limited data exist on the microbial composition of sourdough bread, likely due to the impact of heat during baking, with only one study showing a gene copy number of 7 to 10 log gene copies/gram of sourdough bread [163].

The mechanisms through which sourdough bread may confer health benefits is primarily through the impact that the sourdough process has on the nutritional content of bread. For example, the sourdough process can lower the bread’s content of non-digestible oligosaccharides fructans and raffinose (types of FODMAPs), resulting in the bread being better tolerated by patients with IBS [11,164]. This change in the carbohydrate content occurs due to the degradation of oligosaccharides by the sourdough microorganisms, especially the yeasts *Saccharomyces cerevisiae* and *Kluyveromyces marxianus* [165]. Sourdough and its constituent microorganisms have also been suggested to exhibit anti-microbial, anti-hypertensive, and cholesterol lowering properties, however these are based on in vitro studies examining the impact of sourdough-extracted bacteria, rather than of baked sourdough bread [166,167,168].

The effect of sourdough bread on the gut microbiota has been assessed in vitro and in vivo. The impact of sourdough wheat breads fermented for different lengths of time on the human gut microbiota was assessed using in vitro batch cultures with stool samples from 3 patients with IBS and 3 healthy donors [169]. A significant increase in bifidobacteria was shown in the healthy control samples after addition of sourdough bread fermented for 8 h compared to a non-fermented bread. Significant decreases in δ-Proteobacteria and Gemmatimonadetes was shown after inoculation of sourdough bread fermented for 8 hours in both patients with IBS and healthy donors, compared to baseline (prior inoculation) [169]. In addition, sourdough bread that was fermented for 8 h resulted in significantly lower gas production after 15 h of inoculation of IBS samples, compared to a non-fermented bread and bread fermented with yeast for 16 h. The authors suggested this may indicate that this sourdough bread was fermented more slowly by the gut microbiota [169]. In vivo, however, a randomised cross-over study in 20 healthy adults showed no significant differences in stool microbiota composition when consuming 145 g wholegrain wheat sourdough bread per day for 1 week, compared to 110 g white wheat bread, with the microbiota remaining resilient throughout both bread interventions [170].

### Sourdough Bread in Gastrointestinal Health and Disease

Several studies have examined the impact of sourdough bread in gastrointestinal function and disorders. In a double-blind, cross-over RCT, 17 healthy adults were randomised to consume a single meal of 2 sourdough croissants or 2 brewer’s yeast croissants, followed by magnetic resonance imaging analysis of gastric emptying [171]. The total gastric volume was significantly reduced by 11% and hydrogen production by 30% following sourdough croissants compared to brewer’s yeast croissants [171]. Abdominal discomfort, bloating and nausea were significantly milder, suggesting sourdough croissants are better tolerated than brewer’s yeast croissants [171]. In addition, a very small randomised cross-over trial of 7 participants reporting minor gastrointestinal symptoms showed a significantly different exhaled breath volatile organic compound profile following sourdough rye bread compared to wheat bread enriched with bioprocessed rye bran, however the impact on gastrointestinal symptoms was not measured. This suggests that the potential health effects of sourdough rye bread may indeed be mediated by the gut microbiota [172] (Table 4).

The utility of adjusting the sourdough process to modify the nutritional content of bread was shown in a double-blinded, cross-over RCT in 87 patients with IBS that examined the impact of a sourdough rye bread prepared using a specific sourdough system that lowers the FODMAP content of the bread versus a traditionally-made sourdough bread in gastrointestinal symptoms [11]. This study showed a significantly lower breath hydrogen level, and significantly milder flatulence, abdominal cramps, rumbling and total gastrointestinal symptoms following a 4-week consumption of a low FODMAP sourdough rye bread, compared to a traditional sourdough rye bread [11]. In contrast, a pilot study of 26 people with IBS who were randomised to a 7-day consumption of sourdough wheat bread (low in FODMAPs) or yeast-fermented wheat bread showed that not only were there no differences for any gastrointestinal symptom or inflammation markers, but, unexpectedly, symptoms of tiredness, decreased alertness and joint symptoms were significantly worse in the sourdough wheat bread group, compared to the yeast-fermented wheat bread. It is important to note however that this was a pilot study with a small sample size that used non-validated questionnaires to assess symptoms [164].

Sourdough microorganisms also contain enzymes (e.g., proteases) that hydrolyse proteins, such as gluten. As a result, studies have attempted to study the impact of sourdough bread, fermented by specific combinations of bacteria and yeasts that hydrolyse gluten into amino acids [175], in patients with coeliac disease. Eight paediatric patients with coeliac disease with normal values for total serum IgA were asked to consume 200 g/day of sweet baked products made from fermented wheat flour (containing < 10 ppm residual gluten), while continuing their usual gluten-free diet, for 60 days; six out of eight patients completed the trial, all of whom had normal IgG and IgA-AGA and IgA-tTG antibodies values at the end of the intervention [173]. Similarly, a study in 20 patients with coeliac disease who were randomised to consume sourdough wheat bread (fermented with sourdough lactobacilli and yeast proteases to hydrolyse gluten) or traditional wheat bread for 3 days, found no significant changes in INF-γ secretion in patients consuming the fermented wheat bread, whereas the consumption of traditional wheat bread mobilised INF-γ-secreting cells in blood [174]. However, the duration of the intervention was only 3 days, which is not long enough to assess its impact on inflammatory markers related to coeliac disease. An important consideration should be whether sourdough bread is indeed gluten-free. An in vitro study showed that the level of gluten degradation depends on the strain of the sourdough microorganisms used, and that fermentation of wheat flour does not sufficiently decrease transglutaminase 2 binding sites on gliadin [176]. Therefore, not all gluten is hydrolysed during fermentation and, therefore, sourdough bread made from gluten-containing flour is not considered safe for consumption in coeliac disease.

To conclude, a small study showed no impact of sourdough bread on the stool microbiota composition, while preliminary evidence of the impact of sourdough bread in managing gastrointestinal symptoms stems from low-quality studies, with study sample sizes ranging from 7 to 26 participants. Further high-quality adequately powered studies are needed in order to establish the impact of sourdough bread on gastrointestinal health.

## 8. Conclusions

In summary, there is only very limited evidence on the effectiveness of most fermented foods in gastrointestinal health, with the majority of studies being of low quality. Kefir is the fermented food most commonly investigated in terms of its impact in gastrointestinal health, with evidence suggesting it may be beneficial for lactose malabsorption and *H. pylori* eradication. No human studies have been conducted on the impact of kombucha, tempeh and kimchi in gastrointestinal health. It is worth noting the difficulty in undertaking and replicating fermented food studies given the significant variability of cultures and ingredients present even within food categories, which may partly explain heterogeneous findings. To conclude, there is insufficient evidence to determine the impact of fermented foods in gastrointestinal health and disease.

## Figures and Tables

**Table 1:** Description and microbial content of common fermented foods.

Name	Description	Region of Origin	Source of Microorganisms	Microorganisms Identified in Final Product *
**Kefir**	Fermented milk beverage	Caucasus	Starter culture	*Lactobacillus kefiri*, *Lactobacillus paracasei*, *Lactobacillus parabuchneri*, *Lactobacillus casei*, *Lactobacillus lactis*, *Lactococcus lactis*, *Acetobacter lovaniensis*, *Kluyveromyces Lactis*, *Saccharomyces cerevisiae*
**Kombucha**	Fermented tea beverage	China	Starter culture	*Komagataeibacter xylinus*, *Saccharomyces cerevisiae*, *Zygosaccharomyces bailii*. *Brettanomyces bruxellensis*, *Acetobacter pasteurianus*, *Acetobacter aceti*, *Saccharomyces cerevisiae*, *Zygosaccharomyces bailii*, *Brettanomyces bruxellensis*, *Acetobacter xylinum*, *Zygosaccharomyces* spp., *Acetobacter*, *Gluconacetobacter*
**Sauerkraut**	Fermented cabbage	China	Spontaneous	*Lactobacillus sakei*, *L. plantarum*, *Candidatus accumulibacter phosphatis*, *Thermatoga* spp., *Pseudomonas rhizosphaerae*, *L. hokkaidonensis*, *L. rhamnosus*, *Leuconostoc carnosum*, *Clostridium saccharobutyrilicum*, *Rahnella aquatillis*, *Citrobacter freundii*, *Enterobacter cloacae*, *Bifidobacterium dentium*, *Enterococcus faecalis*, *Lactobacillus casei*, *Lactobacillus delbrueckii*, *Staphylococcus epidermidis*, *Lactobacillus curvatus*, *Lactobacillus brevis*, *Weissella confusa*, *Lactococcus lactis*, *Enterobacteriaceae*, *Leuconostoc* spp., *Yarrowia brassicae*
**Tempeh**	Fermented boiled and dehulled soybeans	Indonesia	Starter culture (*Rhizopus oligoporus*)	*Enterococcus faecium*, *Rhizopus oryzae*, *Rhizopus oligoporus*, *Mucor indicus*, *Mucor circinelloides*, *Geotrichum candidum*, *Aureobasidium pullulans*, *Alternaria alternata*, *Cladosporium oxysporum*, *Trichosporon beigelii*, *Clavispora lusitaniae*, *Candida maltosa*, *Candida intermedia*, *Yarrowia lipolytica*, *Lodderomyces elongisporus*, *Rhodotorula mucilaginosa*, *Candida sake*, *Hansenula fabiani*, *Candida tropicalis*, *Candida parapsilosis*, *Pichia membranefaciens*, *Rhodotorula rubra*, *Candida rugosa*, *Candida curvata*, *Hansenula anomola*
**Natto**	Fermented soybean	Japan	Starter culture (*Bacillus subtilis* natto)	Data not available
**Miso**	Fermented soybean paste	Japan	Starter culture (*Aspergillus oryzae*)	*Bacillus subtilis*, *Bacillus amyloliquefaciens*, *Staphylococcus gallinarum*, *Staphylococcus kloosii*, *Lactococcus* sp. GM005
**Kimchi**	Fermented vegetable dish	Korea	Spontaneous, Addedcommercially	*Leuconostoc gasicomitatum*, *Leuconostoc gelidum*, *Leuconostoc mesenteroides*, *Weissella koreensis*, *Weissella confuse*, *Lactobacillus sakei*, *Lactobacillus plantarum*, *Lactobacillus curvatus*, *Trichosporon domesticum*, *Trichosporon loubieri*, *Saccharomyces unisporus*, *Pichia kluyveri*
**Sourdough bread**	Bread made from longer ferment	Middle East and Europe	Spontaneous or backslopping	Data not available

* Data taken from [2,14,15,16,17,18,19,20,21,22,23,24,25,26,27,28,29,30,31,32,33].

**Table 2:** Summary of interventions studies investigating the impact of kefir in gastrointestinal health and disease.

Study	Study Design	Study Population	Intervention	Control	Duration	Gut Microbiota	Other Findings
**Ino et al., 2015** **[58]**	Non-randomised, cross-over controlled intervention	Constipation, *n* = 11	6 g/day lyophilized kefir. 3 g/day lactose in last 40 day of treatment period	6 g/day powdered milk (baby-formula)	3 months	Not reported	Only three of the 11 participants experienced “more frequent BM without laxative use”. Summary descriptive statistics not shown.
**Maki et al., 2018** **[59]**	Non-randomised, cross-over intervention study	Constipation (hospitalised), *n* = 42	6 g/day of lyophilized kefir	6 g/day powdered milk	12 weeks each period	Not reported	No difference in laxative use between kefir and control groups (7.5 times/3 months *vs* 8.1 times/3 months; *p* = 0.35). No difference in number of people who did not require laxatives. No difference in stool consistency/volume.
**Turan et al., 2014** **[60]**	Non-randomised, uncontrolled intervention study	Functional constipation, *n* = 20	500 mL/day kefir	-	4 weeks	Not reported	Increased stool frequency at follow-up compared to baseline (median 2 BM/week *vs* 5 BM/week; *p* < 0.001). Fewer people with hard stools at follow-up compared to baseline (12/20 *vs* 6/20; *p* = 0.014). Improvement in bowel satisfaction scores (*p* = 0.001). Reduction in gut transit time in participants with slow gut transit time at baseline (*p* = 0.013). No change in straining or laxative use. No major adverse events.
**Bekar et al., 2011** **[61]**	Double-blind RCT	Dyspepsia and *H. pylori* infection, *n* = 85	500 mL/day kefir	250 mL/day milk	2 weeks	Not reported	Higher *H. pylori* eradication rate in kefir *vs* control (78% *vs* 50%; *p* = 0.026). Lower occurrence of diarrhoea (relative risk RR = 0.48; *p* = 0.001), headache (RR=0.17; *p* = 0.008), nausea (RR = 0.47; *p* = 0.029), and abdominal pain (RR = 0.38; *p* < 0.001).
**Hertzler et al., 2003** **[57]**	Cross-over RCT	Lactose malabsorption, *n* = 15	1) 508 mL/day plain kefir 2) 519 g/day raspberry flavoured kefir (equivalent to 20 g lactose)	3) 407 mL/day low fat cow’s milk 4) 378 g/day plain yoghurt (equivalent to 20 g lactose)	Acute 5-day study, each treatment followed by an 8 h breath H_2_ test	Not reported	Higher breath H_2_ AUC in milk compared with plain kefir (*p* = 0.001), plain yogurt (*p* = 0.001), or flavoured yogurt (*p* = 0.005). Higher breath hydrogen AUC in flavoured kefir compared to plain yogurt (*p* = 0.043) or plain kefir (*p* = 0.008). No difference in breath hydrogen AUC between flavoured kefir and milk (*p* = 0.425) or flavoured yogurt (*p* = 0.331). No difference in flatulence severity and frequency, diarrhoea and abdominal pain.
**Merenstein et al., 2009** **[62]**	Double-blind RCT	Antibiotic-associated diarrhoea, *n* = 125	75 mL/day to 150 mL/day kefir	Heat-treated kefir	2 weeks	-	No difference in rates of diarrhoea (relative risk 0.82, 95% CI 0.54–1.43).
**Yilmaz et al., 2018** **[56]**	RCT	Inflammatory bowel disease, *n* = 45 (15 UC, 10 Crohn’s disease)	400 mL/day kefir	No kefir	4 weeks	UC: No difference in change of *Lactobacillus* Crohn’s: Higher change in *Lactobacillus* in kefir compared to control (3.4% log_10_ *vs* –0.6% log_10_; *p* = 0.024).	UC patients: No difference in change of blood haemoglobin concentration Crohn’s disease: Higher change in blood haemoglobin in the kefir group compared to control (0.08% *vs* −0.01%; *p* = 0.029) No difference in change of blood CRP between the kefir and control group

AUC area under the curve; BM bowel movements; RCT randomized controlled trial; UC ulcerative colitis.

**Table 3:** Summary of interventions studies investigating the impact of sauerkraut, soy products and kimchi in gastrointestinal health and disease.

Study	Fermented Food	Study Design	Study Population	Intervention	Control	Duration	Gut Microbiota	Other Findings
**Fujisawa et al., 2006** **[104]**	Natto/miso	Uncontrolled open-label study	Healthy, *n* = 8	200 mL miso soup containing 50 g Natto per day	-	2 weeks	Following natto-containing soup: Higher *Bifidobacteria* and Bacilli, Lower Enterobacteriaceae, Higher acetic acid and propionic acid (all *p* < 0.05)	-
**Kil et al, 2004** **[105]**	Kimchi	Non-randomised trial	*H. pylori* infection, *n* = 6	300 g of kimchi	60 g of kimchi	4 weeks	Increased *Lactobacillus* (p = 0.0003) and *Leuconostoc* (*p* = 0.0004)	*H. pylori* not eradicated in any participants (*p* = 0.944). Lower stool pH (*p* = 0.0001), β-glucuronidase (*p* = 0.0065) and β-glucosidase (*p* = 0.0001) activity
**Mitsui et al., 2006** **[106]**	Natto	Controlled trial	Infrequent bowel movements, *n* = unknown	50 g/day Natto (*Bacillus subtilis* K-2, 3.8 × 10^9^ CFU)	50 g/day boiled soybeans	2 weeks	Following Natto compared to control: Increased ratio of stool *Bifidobacteria*:total bacteria	Following Natto compared to control: Higher number of bowel movements. Higher number of days with bowel movements Higher stool quantity
**Nielsen et al., 2018** **[18]**	Sauerkraut	Randomised, double-blind controlled trial	Irritable bowel syndrome, *n* = 58	75 g/day unpasteurised sauerkraut containing LAB	75 g/day pasteurised sauerkraut	6 weeks	No significant effects of either unpasteurised or pasteurised sauerkraut on microbiota composition	Lower IBS-SSS score following both unpasteurised (*p* = 0.003) and pasteurised (*p* = 0.04) sauerkraut No difference in change in IBS-SSS between groups

LAB, lactic acid bacteria; IBS-SSS Irritable Bowel Syndrome Severity Scoring System.

**Table 4:** Summary of interventions studies investigating the impact of sourdough bread in gastrointestinal health and disease.

Study	Study Design	Study Population	Intervention	Control	Duration	Other Findings
**Korem et al., 2017** **[170]**	Randomised crossover trial	Healthy, *n* = 20	145 g sourdough wholegrain wheat bread	110 g white wheat bread	1 week	Significant interpersonal variability in glycaemic responses Baseline microbiome could predict type of bread that results in lower glycaemic response in each participant
**Polese et al., 2018** **[171]**	Double-blind, cross-over RCT	Healthy, *n* = 17	2 sourdough croissants	2 brewer’s yeast croissants	Single study day	11% decrease in gastric volume AUC 3 h post-consumption (*p* = 0.02) 30% lower hydrogen production during the 4 h post-consumption (*p* = 0.03) Milder abdominal discomfort (*p* = 0.002), bloating (*p* = 0.001) and nausea (*p* = 0.004)
**Raninen et al., 2017** **[172]**	Randomised cross-over trial	Minor gastrointestinal symptoms, *n* = 8	6–10 slices/day of sourdough wholegrain rye bread	6–10 slices/day of wheat bread enriched with bioprocessed (fermented) rye bran	4 weeks	Significant difference in exhaled breath volatile organic compound profile between groups in fasting state (*p* = 0.026). No difference was shown at 30, 60 and 120 min after a standardised meal
**Laatikainen et al., 2016** **[11]**	Randomised, double-blinded, cross-over trial	Irritable bowel syndrome, *n* = 87	7–8 slices/day low FODMAP sourdough rye bread	7–8 slices/day traditional sourdough rye bread	4 weeks	Lower breath H_2_ in low FODMAP rye bread group compared to traditional rye bread (median AUC 53 ppm *vs* 73; *p* = 0.01) Milder flatulence (*p* = 0.04), abdominal cramps (*p* = 0.01), rumbling (*p* = 0.001) and total symptoms (*p* = 0.02) No difference in IBS-SSS (*p* = 0.40). Lower weight in low FODMAP rye bread compared to traditional rye bread (mean difference −0.5 kg, 95% CI –0.9 –0.0; *p* = 0.03)
**Laatikainen et al., 2017** **[164]**	Double-blinded RCT	Irritable bowel syndrome with subjective wheat intolerance, *n* = 26	6 slices/day sourdough wheat bread (fermentation time > 12 h)	6 slices/day yeast-fermented wheat bread (fermentation time approx. 2 h)	7 days	No difference in gastrointestinal symptoms or markers of low-grade inflammation. Worse symptoms of tiredness (*p* = 0.01), joint symptoms (*p* = 0.03) and “decreased alertness” (*p* = 0.003)
**Di Cagno et al., 2010** **[173]**	Non-randomised, uncontrolled study	Coeliac disease, *n* = 8	200 g/day baked products with sourdough wheat flour (10 g hydrolysed gluten)	None	60 days	All patients had normal IgG and IgA-AGA and IgA-tTG antibodies values at the end of the intervention period
**Mandile et al., 2017** **[174]**	RCT	Coeliac disease, *n* = 20	Sourdough wheat bread (fermented with lactobacilli and yeast)	Traditional wheat bread	3 days	No increase in INF-γ secretion Mobilisation of INF-γ secreting cells in the blood following traditional wheat bread

IBS-SSS Irritable Bowel Syndrome Severity Scoring System; RCT, randomized controlled trial.
